# Homicide Profiles Based on Crime Scene and Victim Characteristics

**DOI:** 10.3390/ijerph16193629

**Published:** 2019-09-27

**Authors:** María del Mar Pecino-Latorre, María del Carmen Pérez-Fuentes, Rosa María Patró-Hernández

**Affiliations:** 1 Department of Psychology, University of Almería, 04120 Almería, Spain; marpecino@gmail.com; 2 Department of Psychology, Faculty of Psychology, Universidad Politécnica y Artística del Paraguay, Asunción 1628, Paraguay; 3 Department of Psychology, University of Murcia, 30100 Murcia, Spain; rosapatro@um.es

**Keywords:** homicide, criminal profiling, crime scene behavior, victim, classification tree

## Abstract

One of the current trends in homicide research includes developing works based on scientific study and empirical evidence, which offer conclusions that can be used in an operational manner during police investigations. The objective of this study was to identify homicide characteristics from behaviors carried out on the crime scene and victim characteristics associated with those of the perpetrators of these crimes in Spain. The sample consisted of 448 homicide cases from the database of the Homicide Revision Project led by the Office of Coordination and Studies of the Secretary of State and Security. After creating six classification tree models, it was found that the modus operandi of the aggressor and the victim characteristics may permit hypothesizing about the demographic characteristics of the perpetrator (gender, age, and country of origin), his/her criminal record, and the type of relationship with the victim. Furthermore, the importance of the study of victimology during a criminal investigation is highlighted, as it may indirectly offer information about the potential perpetrator. The findings of this study suggest that criminal profiling contributes notably to the decision-making process to establish more rigorous suspect prioritization, improve the management of human resources and materials, and increase the efficiency of criminal investigations.

## 1. Introduction

Homicide investigation has gained considerable academic and professional interest, not only because homicide is the most violent of the criminal behaviors but also because it impacts on the psychosocial, political, and socio-economic aspects of a country [1]. In fact, the United Nations Office on Drugs and Crime (UNODC) has declared that homicide is a powerful indicator for determining a country’s level of violence and safety [2].

Currently, there is a widespread interest in the study of homicide, not only by international organizations, such as the UNODC and the World Health Organization [2,3,4,5], but also by the academic world, where numerous studies have been conducted in an attempt to extend the knowledge of this criminal phenomenon from diverse perspectives (socio-cultural development factors, psychopathological, neuropsychological) [6,7,8,9]. 

Over recent decades, investigative trends have evolved significantly, especially with regards to the study focus, ways to perceive homicides, and methodology and statistical procedures used. In this sense, homicide is considered to be an extremely complex phenomenon, since it is a criminal category that includes distinct variants with dynamic characteristics and specific psychological processes, as well as distinct characteristics related to the criminal act and its participants [10,11,12,13]. Therefore, it has been suggested that each form of homicide be explored independently, examining specific aspects instead of seeking out generalized associations [14].

On the other hand, although traditional statistical methods focusing on mere descriptions of the phenomena continue to be used in investigations, the use of more sophisticated statistical analysis methods such as multivariate techniques has become more popular. One of the advantages of these statistical procedures is that they consider the combined relationships between all elements that are linked to homicides, thereby ensuring a better understanding of the phenomena and permitting useful conclusions to be reached [6,15,16,17]. In this way, studies focusing on prediction stand out, specifically those focusing on the applied nature of criminal profiling during police investigations, which has been conceived as a supplementary technique to help in the identification and arrest of criminals [18,19,20].

In this context, the “Behavioral Investigative Adviser” has promoted the professionalization of the profiler in police investigations and has prompted the development of empirical works which, from a scientific approach and on the basis of empirical evidence, offer information that can be used operationally [21]. However, this information is not binding, as it is presented in probabilistic terms [22,23,24].

This approach does not focus on the traditional view of the profiler as one who predicts the unknown criminal’s personality or psychological traits. Rather, it is based on elements of the crime that allow for the hypothesizing of potential characteristics that help make decisions to establish more rigorous suspect prioritization and, therefore, will increase the efficiency of the police investigation. For example, some of the perpetrator’s characteristics that are of greatest interest are related to socio-demographic variables (gender, age, country of origin), criminal record history, and the type of relationship existing with the victim [24,25,26]. 

Some authors have focused on analyzing the differences in modus operandi according to the perpetrator’s gender. For example, Sea, Youngs, and Tkazky [27] found that men are more likely to kill women and people that they know, whereas women are more likely to kill individuals from the family environment. Similarly, Häkkänen-Nyholm et al. [28] revealed the trend to use firearms by men, unlike women who tend to use suffocation methods on their victims, who are from the family environment and usually minors. Similar results were found by Fujita et al. [29], suggesting that there is a greater probability of men being the perpetrators when firearms are used. In this way, Chan, Heide, and Beauregard [30] found different trends in the murder weapons used by male and female sexual homicide offenders. Specifically, Chan and Frei [31] demonstrated that the choice of the murder weapons was determined by the victim age and gender.

Similarly, it has been found that, as the aggressor’s age increases, there is a greater tendency to use bladed weapons and blunt objects [32]. Likewise, Khoshnood and Väfors Fritz [33] revealed that perpetrators between the ages of 37 and 59 are associated with non-premeditated-approach methods, thus these homicides tend to be triggered by arguments. Also, Fujita et al. [29] demonstrated that there is a greater probability that the perpetrator will be over the age of 55 when the victim is female and older than 65.

Few studies have examined whether or not differences exist in how the homicides are carried out based on the perpetrator’s country of origin. Soria-Verde, Pufulete, and Álvarez-Llaberia [34] revealed that no significant differences exist between Spanish and immigrant homicides, with regards to the modus operandi or the victim characteristics, only finding that prior arguments were more predominant in the homicides committed by immigrants.

Similarly, perpetrators with a criminal record tend to use more precautionary methods to avoid being identified, such as moving and hiding the cadaver, physically distancing themselves from the crime scene, or setting the crime scene on fire to eliminate physical evidence [35,36,37,38]. Also, they are more frequently associated with robberies and sexual aggressions that occur during the course of the homicide [29,35]. Likewise, perpetrators with a violent criminal record tend to commit homicides in outdoor locations and tend to have male victims as well as victims aged between 19 and 35 and over the age of 56 [39,40]. 

Finally, it has been shown that in the case of perpetrators who previously knew their victims, there tends to be a greater number and seriousness of injuries [41], with the homicides tending to take place indoors and most often using bladed weapons or blunt objects [32,42]. In addition, they tend to be triggered by an argument [33,43]; similarly, the victims are usually male and between the ages of 15 and 24 [44]. Also, homicides committed in the family setting are associated with victims who are usually minors and elderly individuals, and suffocation and poisoning predominate as the homicide methods used [37,39,40,44]. In homicides taking place between couples, the victims tend to be females, aged between 19 and 50, means are commonly used to control the victim, and bladed weapons and blunt objects are most often used [39,44]. Finally, homicides in which the victims are unknown individuals tend to take place during the course of other criminal activities (e.g., robberies, sexual aggressions) and in outdoor locations. In these cases, there tends to be a greater forensic awareness by the perpetrator [32,43,44,45].

In the reviewed literature, the usefulness of inferring the perpetrator’s characteristics on the basis of an analysis of the homicide elements has been shown [46]. Specifically, the investigation has indicated that the type of weapon used and/or the method used to commit the homicide, the location where the homicide takes place, and the method of approach used, among others, are aspects that offer information on the socio-demographic characteristics of the aggressor, the criminal record history, and the victim–perpetrator relationship [27,29]. Likewise, the study of the victim characteristics is a key element of analysis that may provide relevant information so as to determine the potential characteristics of the aggressor [36,47]. Therefore, the objective of this work was to determine which characteristics of the homicide, of the behaviors carried out at the crime scene, and of the victims are associated with the characteristics of the simple homicide perpetrator in Spain.

## 2. Methods 

### 2.1. Participants

An initial sample of 684 homicide cases registered in Spain between 2010 and 2012 (both inclusive) was used, discarding cases of multiple homicide (more than one perpetrator and/or victim) (*n* = −213), those carried out by minors (*n* = −13), those that were not solved by the police (*n* = −1) and those that did not contain information (*n* = −9). Finally, the study sample included a total of 448 homicides, 89% (*n* = 388) of which were carried out by individuals that knew their victim well or that did not have a close relationship with the victim (including strangers), and 11% (*n* = 48) of which were linked to the committing of another criminal activity. Of the entire sample, 90.8% (*n* = 407) of the perpetrators were male and 9.2% (*n* = 41) were female, aged 41.35 (SD = 15.24) and 39.24 (SD = 14.63) on average, respectively. 

### 2.2. Procedure

The Office of Coordination and Studies of the Secretary of State and Security, in collaboration with the national security forces (Civil Guard and National Police force) and several Spanish universities developed and coordinated the Homicide Revision Project. The parties responsible for this project requested the relevant reports from the corresponding police departments and created a database to permit information collection. Next, specialized training was received on how to carry out the data dump procedure, ensuring confidentiality and ethical data treatment. 

Having completed the database, and with the authorization of the responsible party of the Office of Coordination and Studies of the Secretary of State and Security, the database was provided in order to carry out this study. 

Then, the sample was selected, not on the basis of a probabilistic procedure but via convenience sampling, in which all available cases were analyzed, considering the exclusion criteria described above. Despite the fact that the sample would not be representative of all of the homicides taking place in Spain, it was representative of those simple homicides that had been solved by the police.

Finally, a data-cleaning process was carried out in order to thoroughly analyze the quality of the information and to prepare the matrix for statistical analysis.

### 2.3. Data Analysis

#### Classification Trees

In this study, Classification and Regression Trees (CART) were used, with R statistics software (package ‘rpart’) [48]. This is one of the most efficient multi-variate classification techniques of machine learning, belonging to the family of Decision Trees, whose purpose is to classify future observations on the basis of a set of rules. For this, through a sequential process, the classification variables that best differentiate the groups in relation to the dependent variable (child nodes) are identified, dividing the dataset recursively until the majority of the cases have been correctly classified into one category (terminal or leaf node) [49].

Since the variables were categorical in this study, classification trees and Gini impurity measure were used as goodness-of-fit criteria to make the data partition. Then, the tree was pruned, using a tenfold cross validation procedure to determine the optimal tree size. In this way, the tree whose complexity parameter minimized the mean error of the cross-validation (*x error*) was selected [16,50].

Thus, a classification tree was created for each of the perpetrator variables and for the type of relationship with the victim (offender’s sex, age, country origin, criminal record, crimes against the person, and victim–offender relationship), considering those variables related to the homicide and the victim characteristics as the classification variables (Table 1). Likewise, the minimum number of observations in the nodes was determined as 20 before partitioning the data and as 6 for the terminal nodes. 

In the tree diagrams, each route, from a root node to a leaf node, represents a decision rule that characterizes a profile according to the dependent variable, such that each model offers a general view of how the relative variables of the homicide and victim characteristics interact and combine so as to discriminate between the perpetrator characteristics.

## 3. Results

Below, the graphical representations obtained from the resulting models for the perpetrator’s gender, age, country of origin, criminal record, and victim–offender relationship are presented, with each node represented as a rectangle, colored according to the majority category of data which is included in it, specifying the probability per class of the observation in the node.

### 3.1. Classification Trees

#### 3.1.1. Classification Model for the Gender of the Perpetrator

**Figure 1:**
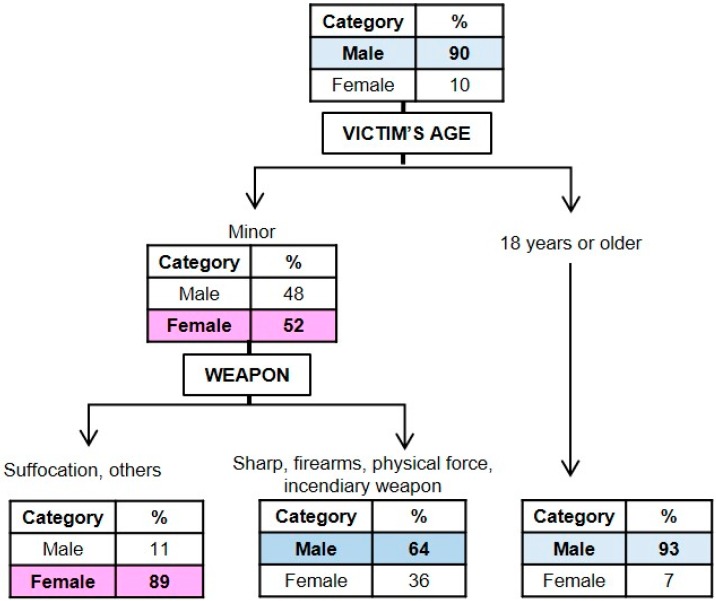
Graphical representation of the classification tree for the perpetrator’s gender.

In Figure 1, a graphical representation is presented of the model obtained for the perpetrator’s gender (*x error* = 0.924). It may be seen that the most relevant variables for identifying the gender of the perpetrator were victim age and homicide weapon or the methods used to commit the homicide. Taking into account that the initial probability of the perpetrator being male was 90% and that of being female was 10%, it is considered that these variables are related to perpetrator gender. In cases in which the victims were minors (<18 years of age), the probability that the perpetrator was female increases from 10% to 52% and if, in addition, suffocation methods or other weapon types were used, there is a greater probability that the homicide was carried out by a female (89%). On the other hand, there is a high probability that the homicide was carried out by a male (93%) if the victim was 18 years of age or older.

#### 3.1.2. Classification Model for the Age of the Perpetrator:

**Figure 2:**
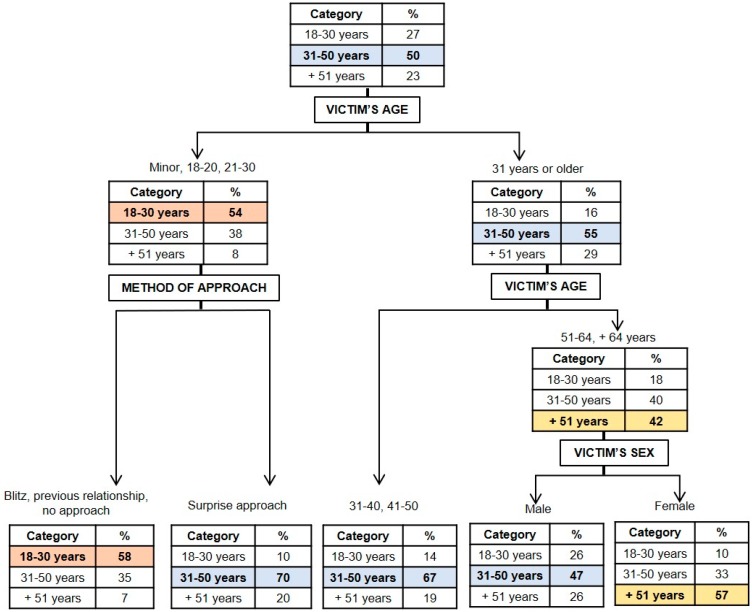
Graphical representation of the classification tree for the perpetrator’s age.

In Figure 2, a graphical representation is presented of the model resulting for the age of the perpetrator (*x error* = 0.875). It may be observed that the age and the gender of the victim, as well as the approach method used by the aggressor, were the most important variables in determining the age of the perpetrator. Taking into account that the initial probability of the perpetrator being between the ages of 18 and 30 was 27%, that of being between 31 and 50 years of age was 50%, and that of being older than 51 was 23%, it was considered that these variables are related to the age of the perpetrator by age bracket. When the victims were minors or are aged between 18 and 30 years, the probability of the perpetrator having the same age as the victim increased from 27 to 54%. If, in addition, the perpetrator approached the victim suddenly, there was a previous relationship between them, or the approach did not take place with the intent to commit a homicide, the probability of the aggressor being aged between 18 and 30 increased to 58%. On the other hand, in cases where there was a surprise approach to the victims that were minors or aged between 18 and 30, the probability that the perpetrator was aged from 31 to 50 years rose from 50% to 70%. Also, when the victims were over the age of 51, the probability that the perpetrator was also over 51 years of age rose from 23% to 42%. If, in addition, the victims were females, this probability reached 57%.

#### 3.1.3. Classification Model for the Country of Origin of the Perpetrator

**Figure 3:**
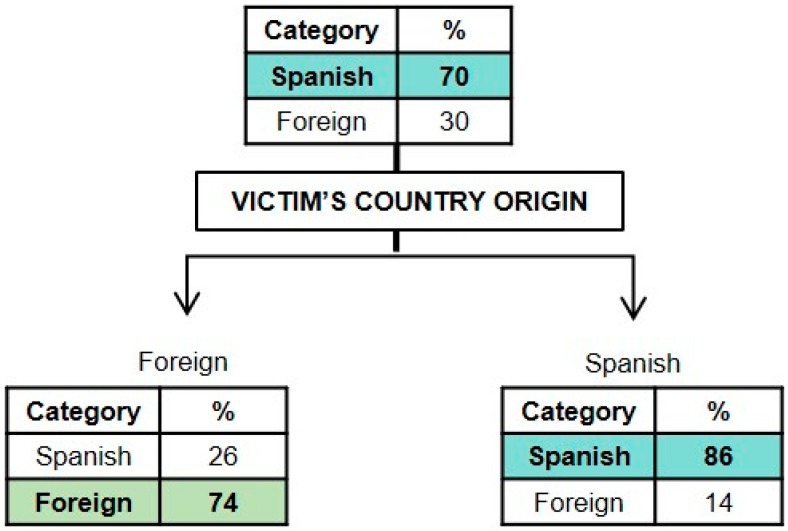
Graphical representation of the classification tree for the perpetrator’s country of origin.

In Figure 3, the graphical representation of the model obtained for the perpetrator’s country of origin is presented (*x error* = 0.567). It may be observed that the victim’s country of origin is the most relevant characteristic associated with the perpetrator’s country of origin. Taking into account that the initial probability of the perpetrator being Spanish was 70% and the probability that he/she was a foreigner was 30%, the probability of the aggressor being a foreigner increased from 30 to 74% when the victim was also a foreigner, just as the probability of the perpetrator being Spanish increases from 70 to 86% when the victim was Spanish.

#### 3.1.4. Classification Model for the Criminal Record History of the Perpetrator

**Figure 4:**
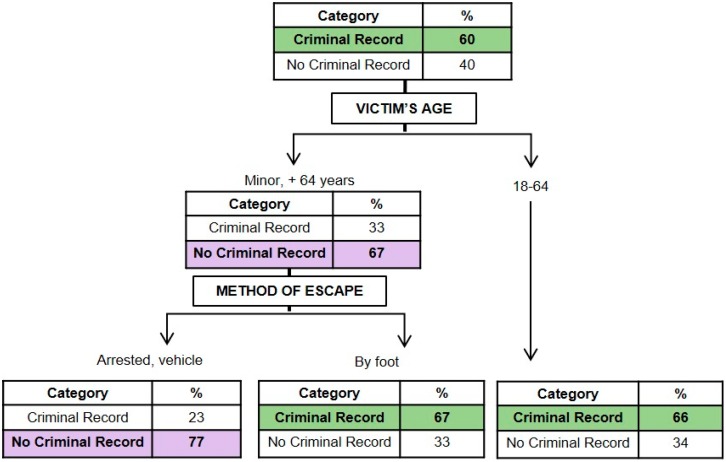
Graphical representation of the classification tree for the perpetrator’s criminal record history.

Figure 4 presents a graphical representation of the model resulting from the perpetrator’s criminal record history (*x error* = 0.872). It may be seen that the age of the victim and the method used to flee were the most important characteristics in determining if the perpetrator had a criminal record or not. The initial probability that the perpetrator had a criminal record was 60%, and that of he/she not having a criminal record was 40%. When the victims were between the ages of 18 and 64, the probability that the perpetrator had a record increased from 60% to 66%. On the other hand, when the victim was a minor or over the age of 64, the probability of the perpetrator not having a record increased from 40% to 67%. If, in addition, if the perpetrator fled from the scene of the crime in a vehicle or was detained at the crime scene, the probability that the perpetrator did not have a record rose (77%). On the other hand, in cases of minor victims or those over the age of 64, when the perpetrator fled the crime scene by foot, the probability that he/she had a record was 67%.

#### 3.1.5. Classification Model for the Criminal Record for Crimes Against Persons of the Perpetrator

**Figure 5:**
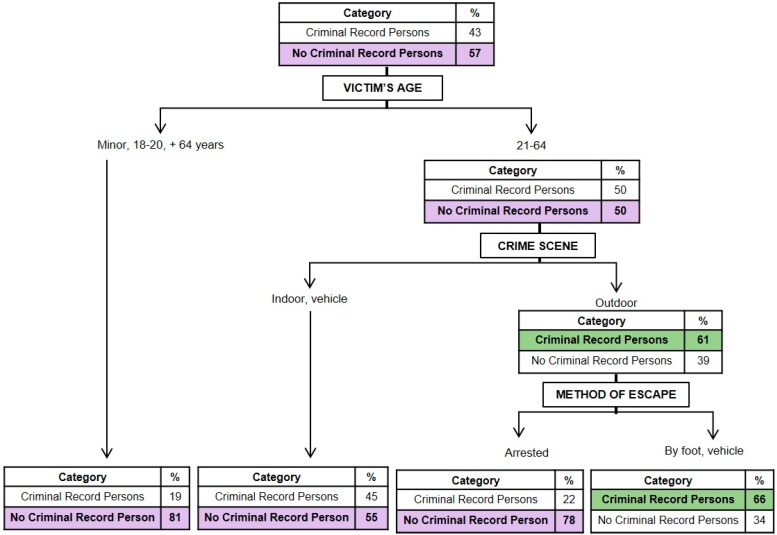
Graphical representation of the classification tree for the perpetrator’s criminal record of crimes against persons.

In Figure 5, a graphical representation is shown of the model obtained for the perpetrator’s criminal record history for crimes against persons (*x error* = 0.931). It may be observed that the age of the victim, the type of place where the homicide took place, and the method of fleeing of the aggressor were the most important variables in determining whether or not the aggressor had a record for crimes against persons, referring to those criminal typologies that suggest violent or intimidating behavior against an individual (crimes against the life, integrity, and freedom of others, including homicide). The initial probability that the perpetrator did not have a record for crimes against persons was 57%. In cases in which the victims were minors or over the age of 64, this probability increased to 81%. On the other hand, when the victims were between the ages of 21 and 64 and the homicide took place outdoors, the probability of the perpetrator having a record rose from 43% to 61%. If, in addition, if he/she fled the crime scene by foot or by vehicle, this probability increased to 66%.

#### 3.1.6. Classification Model for the Type of Relationship Between the Victim and the Aggressor

**Figure 6:**
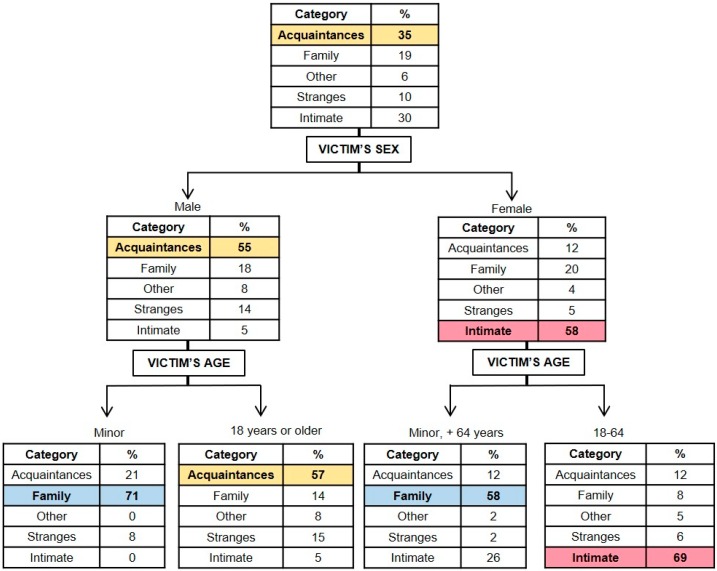
Graphical representation of the classification tree for the type of relationship between the victim and the aggressor. The category “Acquaintances” includes known individuals/neighbors, friends, work/commercial relationships and school relationships. The category “Intimate” includes couples, spouses, ex-partners, separated or divorced.

Figure 6 presents the graphical representation of the model resulting for the relationship between victim and aggressor (*x error* = 0.606). It may be observed that the gender and age of the victim were the variables associated with the type of relationship existing between the victim and the aggressor. Taking into account that the initial probability that they were acquaintances was 35%, that of being family members was 19%, that of having other types of relationship was 6%, that of having no relationship was 10%, and that of having a romantic relationship in the past or present at the time of the event was 30%, it was considered that these variables were related to the type of relationship existing between the aggressor and the victim (except for the categories “other relationship” and “stranger”, which, because of their low prevalence, did not reach the minimum of 20 cases in the classification model). So, when the victim was male and a minor, the probability that the offender was a family member increased from 19% to 71%, unlike the case in which the victim was a male over the age of 18, in which case, there was a greater probability that the perpetrator was an acquaintance of the victim (57%). On the other hand, when the victim was a female between the ages of 18 and 64, the probability that the perpetrator was her romantic partner or ex-partner increased from 30 to 69%.

## 4. Discussion

The results obtained in this study are consistent with the central postulate of criminal profiling, that is, on the basis of homicide elements (crime scene, modus operandi, victim characteristics), it is possible to hypothesize about the potential characteristics of the perpetrator, which helps make decisions to establish more rigorous suspect prioritization [18,21].

On the basis of the socio-demographic characteristics of the perpetrators, the results indicate that there is a greater probability that the perpetrator will be female when the victim is a minor and when suffocation methods are used to commit the homicide. Similarly, prior studies have shown that females are more associated with intra-family homicides and suffocation methods, with the victims being mainly minors (filicide) [27,28,31]. Likewise, in accordance with previous works, the results indicate that men are more likely to use firearms and their own body strength to kill their victims [28,29]. In this way, the results coincide with those of prior studies that showed that the type of murder weapon used by males and females is partially influenced by the victims and their characteristics [30].

As for the age of the perpetrator, we are unaware of prior studies that identify the homicide and victim characteristics and permit the discrimination of perpetrators aged between 18 and 30 and 31 and 50, except for the study by Khoshnood and Väfors Fritz [33], which revealed that homicides carried out by perpetrators between the ages of 37 and 59 tend to be triggered by an argument, without evidence of premeditation. These results are contradictory to the results of this study. The reasons of this difference may be that Khoshnood and Väfors Fritz [33] included a lower number of offenders in their study than were included in this study and that we solely focused on homicide and not on attempted murder/manslaughter, which was included in their study. Likewise, the results obtained indicate that it is more likely for the perpetrator to be over 51 years of age when the victim is in the same age bracket and is female. These results are similar to those found in the work of Fujita et al. [29], in which it was established that the age and gender of the victim are determinant for inferring the age of the perpetrator, suggesting that it is more likely for the perpetrator to be 55 or older when the victim is female and over 65. 

With regards to the country of origin of the perpetrator, the results suggest that there is a greater probability that the aggressor is Spanish when the victims also are, just as it is more likely for the perpetrator to be a foreigner when the victim is also a foreigner, from the same country as the perpetrator’s. Given that we have not been able to find past studies with an international scope that consider whether it is possible to infer the country of origin of a perpetrator on the basis of how the homicide occurs and of the victim characteristics, our results may be related, to some extent, to the findings of Soria-Verde et al. [34], who established that there were no significant differences in the modus operandi between Spanish and immigrant homicides.

As for the existence of a criminal record in general and against persons for the perpetrator, the results support past studies that suggest that perpetrators with a criminal record tend to use precautionary measures to avoid identification [29,38]; thus, fleeing by foot or vehicle from the crime scene is considered a form of distancing oneself from the crime committed [36]. Similarly, according to Salfati and Canter [40], perpetrators with a criminal record commit more homicides in outdoor locations, perhaps because in these locations it is more difficult to find incriminating evidence, given meteorological phenomena which often destroy the said evidence. Similarly, the results coincide with those reported in the study by Santtila, Häkkänen, Canter et al. [39], which affirmed that perpetrators with a record of violent crimes and sexual aggressions are more likely to have victims aged between 19 and 35. 

With regards to prior relationships between the victim and the aggressor, the results from this study are consistent with those of Yang and Olafsson [44], which affirm that, when the victims are males and are over the age of 18, the perpetrators are more likely to be acquaintances. Likewise, the results coincide with those of prior studies that suggest that when the victims are minors or elderly individuals, regardless of the gender, homicides tend to be perpetrated by family members [37,39]. Also, the results obtained with regards to adult female victims show a greater probability of the perpetrator being her romantic partner, either present or past, at the time of the crime, similar to the findings obtained in studies from other countries [39,44]. 

This work has certain limitations. First, the conclusions derived from the study cannot be generalized to other types of homicides, since only simple homicides and those with perpetrators over the age of 18 were considered. Second, there is always the likelihood that human error in the initial data coding may have occurred. In addition, the database did not include detailed information on the scene of the crimes, location of injuries, or circumstances in which the cadaver was found, so future studies should include these variables in their classification models to establish a more rigorous decision-making procedure. Third, the classification tree is a technique that is specific to the sample at hand, meaning that if a different sample was used, the results would most likely vary significantly. So, it would be useful to have a greater volume of cases to create predictive models that serve as support during criminal investigations.

As future lines of study, we suggest establishing predictive models with distinct homicide samples (multiple homicides, juvenile homicides). It would also be interesting to use other statistical procedures that are more complex, such as Bayesian networks, since this would help in the creation of criminal profiles, identifying not only probabilistic relations between variables but also causal relations between them. Similarly, it would be very useful to replicate the methodology used in this work for sexual aggressions, since it is quite possible that distinct interactions between variables will be found.

## 5. Conclusions

In this work, it was possible to detail the characteristics of the homicide, the behaviors carried out at the crime scene, and the victim characteristics associated with the socio-demographic characteristics of the perpetrator of simple homicides in Spain, the existence of a criminal record, and the type of relationship between perpetrator and victim. It is important to examine homicides at a multi-variate analysis level, since this criminal phenomenon is quite complex and it is necessary to consider the collective relationships between all of the related components. In this way, an improved understanding of the phenomenon may be acquired, allowing us to reach conclusions that may help to make decisions during a criminal investigation, especially in suspect prioritization tasks. In fact, many criminology and forensic studies have demonstrated their utility for identifying complex interactions between variables that would not be easily detected with other more traditional statistical procedures (e.g., linear models). Another of the advantages is that, unlike other classification methods, the existence of missing values does not interfere with the development of the model, which is quite common when police records are the main source of information.

This work shows that the study of victimology is key during criminal investigations, since these socio-demographic characteristics (gender, age, and country of origin) offer information on potential perpetrators. Furthermore, some authors have recognized that the victim is an extension of the crime scene and, therefore, victim analysis deserves special attention [23,51]. So, this analysis facilitates the crime reconstruction, helps to identify the modus operandi of the perpetrator, and suggests new lines of study that will help reduce the number of suspects.

Together, these results suggest the relevance of creating studies that, based on empirical evidence, contribute to decision-making in order to establish more rigorous suspect prioritization and to improve human resources and materials management. Ultimately, knowledge of how to interpret the modus operandi at homicide scenes and its relation with potential perpetrator characteristics may help reduce the time and economic resources devoted to criminal investigations.

## Figures and Tables

**Table 1:** Variables used in the classification models.

Classification Variables	Target Variables
Crime scene	Offender’s sex
Method of approach	Offender’s age
Method of control	Offender’s country origin
Method of escape	Offender’s criminal record
Homicide weapon	Offender’s criminal record for crimes against persons
Type of weapon	Victim–offender relationship
Located weapon	
Weapon displacement	
Body displacement	
Hidden body	
Forensic awareness	
Staged	
Sexual assault	
Stole	
Arson	
Victim’s sex	
Victim’s age	
Victim’s country origin	

## References

[1] Ministerio del Interior Informe Sobre el Homicidio: España, 2010–2012. http://www.interior.gob.es/documents/642317/1203227/Informe_sobre_el_homicidio_España_2010-2012_web_126180931.pdf/9c01b8da-d1b8-42b9-9ab0-2cf2c3799fb1.

[2] United Nations Office on Drugs and Crime (UNODC) Global Study on Homicide 2013. http://www.unodc.org/documents/gsh/pdfs/2014_GLOBAL_HOMICIDE_BOOK_web.pdf.

[3] United Nations Office on Drugs and Crime (UNODC) International Classification of Crimen for Statistical Proposes, Version 1.0. https://www.unodc.org/documents/data-and-analysis/statistics/crime/ICCS/ICCS_English_2016_web.pdf.

[4] United Nations Office on Drugs and Crime (UNODC) Global Study on Homicide: Gender-related killing of women and girls. https://www.unodc.org/documents/data-and-analysis/GSH2018/GSH18_Gender-related_killing_of_women_and_girls.pdf.

[5] World Health Organization (WHO), United Nations Office on Drugs and Crime (UNODC), United Nations Development Programme, (UNDP) Global Status Report on Violence Prevention 2014. http://www.who.int/violence_injury_prevention/violence/status_report/2014/en/.

[6] Koeppel M.D.H., Rhineberger-Dunn G.M., Mack K. (2015). Cross-national homicide: a review of the current literature. Int. J. Comp. Appl. Crim. Justice.

[7] Botelho M., Gonçalves R.A. (2016). Why do people kill? A critical review of the literature on factors associated with homicide. Aggress. Violent Behav..

[8] Abreu V., Barker E., Bedford R. (2017). Method of homicide and severe mental illness: A systematic review. Aggress. Violent Behav..

[9] Miller L. (2014). Serial killers: II. Development, dynamics, and forensics. Aggress. Violent Behav..

[10] Salari S., Sillito C.L.F. (2016). Intimate partner homicide-suicide: Perpetrator primary intent across young, middle, and elder adult age categories. Aggress. Violent Behav..

[11] Higgs T., Carter A.J., Tully R.J., Browne K.D. (2017). Sexual murder typologies: A systematic review. Aggress. Violent Behav..

[12] Miller L. (2014). Serial killers: I. Subtypes, patterns, and motives. Aggress. Violent Behav..

[13] Ioannou M., Hammond L., Simpson O. (2015). A model for differentiating school shooters characteristics. J. Crim. Psychol..

[14] Ioannou M., Hammond L. (2015). The changing face of homicide research: The shift in empirical focus and emerging research trends. J. Crim. Psychol..

[15] Kivivuori J., Suonpää K., Lehti M. (2014). Patterns and theories of European homicide research. Eur. J. Criminol..

[16] Masías V.H., Valle M.A., Amar J.J., Cervantes M., Brunal G., Crespo F.A. (2016). Characterising the Personality of the Public Safety Offender and Non-offender using Decision Trees: The Case of Colombia. J. Investig. Psychol. Offender Profiling.

[17] Tonkin M., Lemeire J., Santtila P., Winter J.M. (2019). Linking property crime using offender crime scene behaviour: A comparison of methods. J. Investig. Psychol. Offender Profiling.

[18] Canter D.V., Youngs D. (2009). Investigative Psychology: Offender Profiling and the Analysis of Criminal Action.

[19] Kocsis R.N. (2006). Criminal Profiling: Principles and Practice.

[20] Turvey B.E., Turvey B.E. (2012). An Introduction to Behavioral Evidence Analysis. Criminal Profiling: An Introduction to Behavioral Evidence Analysis.

[21] Chan H.C.O., Beauregard E. (2019). Prostitute homicides: A 37-year exploratory study of the offender, victim, and offense characteristics. Forensic Sci. Int..

[22] Alison L.J., Rainbow L. (2011). Professionalizing Offender Profiling: Forensic and Investigative Psychology in Practice.

[23] Sotoca A., González-Álvarez J.L., Halty L. (2019). Perfiles Criminales. Principios, Técnicas y Aplicaciones.

[24] González-Álvarez J.L., Sotoca A., Garrido M.J., Giménez-Salinas A., González J.L. (2015). El perfilamiento en la investigación criminal. Investigación Criminal: Principios, Técnicas y Aplicaciones.

[25] Farrington D.P., Lambert S., Kocsis R.N. (2007). Predicting Offender Profiles from Offense and Victims Characteristics. Criminal Profiling: International Theory Research and Practice.

[26] Turvey B.E., Turvey B.E. (2012). Inferring Offender Characteristics. Criminal Profiling: An Introduction to Behavioral Evidence Analysis.

[27] Sea J., Youngs D., Tkazky S. (2017). Sex Difference in Homicide: Comparing Male and Female Violent Crimes in Korea. Int. J. Offender Ther. Comp. Criminol..

[28] Häkkänen-Nyholm H., Putkonen H., Lindberg N., Holi M., Rovamo T., Weizmann-Henelius G. (2009). Gender differences in Finnish homicide offence characteristics. Forensic Sci. Int..

[29] Fujita G., Watanabe K., Yokota K., Kuraishi H., Suzuki M., Wachi T., Otsuka Y. (2013). Multivariate Models for Behavioral Offender Profiling of Japanese Homicide. Crim. Justice Behav..

[30] Chan H.C.O., Heide K.M., Beauregard E. (2019). Male and Female Single-Victim Sexual Homicide Offenders: Distinguishing the Types of Weapons Used in Killing Their Victims. Sex. Abus..

[31] Chan H.C.O., Frei A.M. (2013). Female Sexual Homicide Offenders: An Examination of an Underresearched Offender Population. Homicide Stud..

[32] Pelletier K.R., Pizarro J.M. (2018). Homicides and Weapons: Examining the Covariates of Weapon Choice. Homicide Stud..

[33] Khoshnood A., Väfors Fritz M. (2017). Offender Characteristics: A Study of 23 Violent Offenders in Sweden. Deviant Behav..

[34] Soria-Verde M.A., Pufulete E.M., Álvarez-Llaberia F.X. (2018). Homicidios en la Pareja: Explorando las Diferencias entre Agresores inmigrantes y españoles. Anu. Psicol. Jurídica.

[35] Thijssen J., De Ruiter C. (2011). Instrumental and Expressive Violence in Belgian Homicide Perpetrators. J. Investig. Psychol. Offender Profiling.

[36] Garrido V., Sobral J. (2008). La Investigación Criminal. La Psicología Aplicada al Descubrimiento, Captura y Condena de los Criminales.

[37] Salfati C.G. (2000). The Nature of Expressiveness and Instrumentality in Homicide: Implications for Offender Profiling. Homicide Stud..

[38] Häkkänen H., Hurme K., Liukkonen M. (2007). Distance patterns and disposal sites in rural area homicides committed in Finland. J. Investig. Psychol. Offender Profiling.

[39] Santtila P., Häkkänen H., Canter D.V., Elfgren T. (2003). Classifying homicide offenders and predicting their characteristics from crime scene behavior. Scand. J. Psychol..

[40] Salfati C.G., Canter D. (1999). V Differentiating Stranger Murders: Profiling Offender Characteristics from Behavioral Styles. Behav. Sci. Law.

[41] Reckdenwald A., Simone S. (2017). Injury Patterns for Homicide Followed by Suicide by the Relationship Between Victims and Offenders. Homicide Stud..

[42] Chan H.C.O., Beauregard E. (2016). Choice of Weapon or Weapon of Choice? Examining the Interactions between Victim Characteristics in Single-victim Male Sexual Homicide Offenders. J. Investig. Psychol. Offender Profiling.

[43] Goodwill A.M., Allen J.C., Kolarevic D. (2014). Improvement of thematic classification in offender profiling: Classifying serbian homicides using multiple correspondence, cluster, and discriminant function analyses. J. Investig. Psychol. Offender Profiling.

[44] Yang R., Olafsson S. (2011). Classification for predicting offender affiliation with murder victims. Expert Syst. Appl..

[45] Sea J., Beauregard E. (2017). An Analysis of Crime Scene Behavior in Korean Homicide. J. Interpers. Violence.

[46] Crabbé A., Decoene S., Vertommen H. (2008). Profiling homicide offenders: A review of assumptions and theories. Aggress. Violent Behav..

[47] Jiménez J. (2012). Manual Práctico del Perfil Criminológico. Criminal Profiling.

[48] Therneau T., Atkinson B. Package: rpart. https://cran.r-project.org/web/packages/rpart/rpart.pdf.

[49] Therneau T., Atkinson E. An Introduction to Recursive Partitioning Using the RPART Routines. https://cran.r-project.org/web/packages/rpart/vignettes/longintro.pdf.

[50] Ngo F.T., Govindu R., Agarwal A. (2015). Assessing the Predictive Utility of Logistic Regression, Classification and Regression Tree, Chi-Squared Automatic Interaction Detection, and Neural Network Models in Predicting Inmate Misconduct. Am. J. Crim. Justice.

[51] Turvey B.E., Freeman J., Turvey B.E. (2012). Forensic Victimology. Criminal Profiling: An Introduction to Behavioral Evidence Analysis.

